# A Plant-Specific Transcription Factor IIB-Related Protein, pBRP2, Is Involved in Endosperm Growth Control

**DOI:** 10.1371/journal.pone.0017216

**Published:** 2011-02-24

**Authors:** Emilie Cavel, Marion Pillot, Dominique Pontier, Sylvie Lahmy, Natacha Bies-Etheve, Danielle Vega, Daniel Grimanelli, Thierry Lagrange

**Affiliations:** 1 Laboratoire Génome et Développement des Plantes, Centre National de la Recherche Scientifique/Institut de Recherche pour le Développement/Université de Perpignan, Perpignan, France; 2 Institut de Recherche pour le Développement, Montpellier, France; Ecole Normale Superieure, France

## Abstract

General transcription factor IIB (TFIIB) and TFIIB-related factor (BRF), are conserved RNA polymerase II/III (RNAPII/III) selectivity factors that are involved in polymerase recruitment and transcription initiation in eukaryotes. Recent findings have shown that plants have evolved a third type of B-factor, plant-specific TFIIB-related protein 1 (pBRP1), which seems to be involved in RNAPI transcription. Here, we extend the repertoire of B-factors in plants by reporting the characterization of a novel TFIIB-related protein, plant-specific TFIIB-related protein 2 (pBRP2), which is found to date only in the Brassicacea family. Unlike other B-factors that are ubiquitously expressed, *PBRP2* expression is restricted to reproductive organs and seeds as shown by RT-PCR, immunofluorescence labelling and GUS staining experiments. Interestingly, *pbrp2* loss-of-function specifically affects the development of the syncytial endosperm, with both parental contributions required for wild-type development. pBRP2, is the first B-factor to exhibit cell-specific expression and regulation in eukaryotes, and might play a role in enforcing bi-parental reproduction in angiosperms.

## Introduction

In eukaryotes, nuclear gene expression is accomplished by three conserved RNA polymerases (RNAP), namely RNAPI, II, and III. These act in association with a set of auxiliary factors, the General Transcription Factors (GTFs), for selective promoter recognition and transcription initiation [1],[2]. Among the GTF, TATA-binding protein (TBP), general transcription factor B (TFIIB) for RNAPII, and TFIIB-related factor (BRF) for RNAPIII are more evolutionarily conserved [1],[2]. For mRNA-type promoters, transcription factor D (TFIID) first binds to the TATA box *via* its TBP subunit, forming an intermediate pre-initiation complex (PIC) that can further be stabilized and correctly oriented by TFIIB [3],[4]. TFIIB also plays a crucial role in the activity of the PIC, showing absolute requirement at all protein-encoding genes for the recruitment of RNAPII and the positioning of the transcription start site [4],[5].

Although the GTFs have long been thought to be ubiquitous, it is now well documented that animals have evolved variants of TBP and TFIIB with more specialized functions. To date, three TBP- and one TFIIB-related factors have been characterized: TBP-related factor 1 (TRF1), which has only been found in *Drosophila* and *Anopheles*; TBP-like factor (TLF; also called TBPL1/TRF2/TRP), widely distributed among metazoans; TBP2 (also called TRF3/TBPL2), present in vertebrates; and BRF-related factor (BRFU), a B-type factor identified in man [6],[7]. Interestingly, in contrast to TBP genes that are ubiquitously expressed, TRF genes exhibit more restricted cell-type specificity, being preferentially expressed in embryos and/or reproductive organs [8],[9]. Consistent with their expression patterns, mouse TLF and TBP2 variants have been shown to be involved in the development of the germ lines [9]–[11].

In order to improve our understanding of the composition and evolution of the basal transcription machinery in plants, we have searched the Arabidopsis databases for putative variants of the conserved GTFs and which possibly have plant-specific functions. This work identified the first plant-specific TFIIB-related protein, pBRP, hereafter pBRP1 [12], whose orthologues are widely expressed among plant species but also in the red alga *Cyanidioschyzon merolae* [13]. Recent functional studies suggested that pBRP1 factors define a third type of B-factor involved in RNAPI transcription in plant cells [13], emphasizing the importance of searching for GTF variants to reveal novel regulatory pathways in plants. In the present work, we have extended our initial study by reporting the identification of an Arabidopsis gene coding for a novel plant-specific variant of a B-type factor. Phylogenetic analysis showed that the corresponding protein, referred to as pBRP2 (plant-specific TFIIB-related protein 2), belongs to the TFIIB family. In contrast to *PBRP1*, which is widely distributed among plant species, *PBRP2* has been identified to date only in the Brassicacea family, suggesting a recent origin. Moreover, our data indicate that *PBRP2* is specifically expressed in reproductive organs and dry seeds. Using a reverse genetic approach, we have demonstrated that pBRP2 is involved in endosperm proliferation.

## Results and Discussion

### Identification of the Arabidopsis *PBRP2* gene

**Figure 1:**
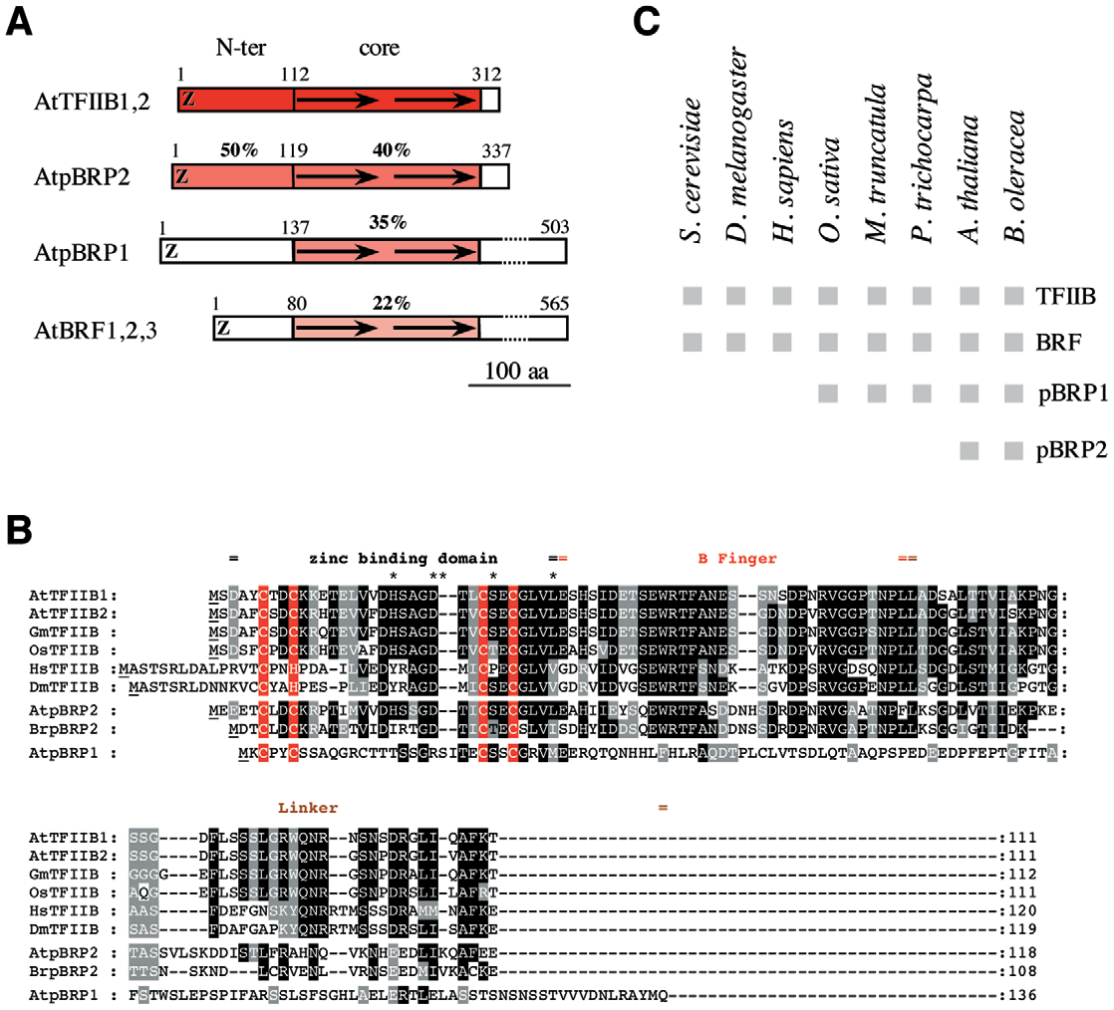
pBRP2 proteins are novel plant-specific members of the TFIIB factor family. A) Schematic structure of the Arabidopsis B-type proteins. The zinc ribbon domain (Z) and imperfect direct repeats (→) of the core domain are indicated, as are the percentage identities. B) Alignment of the N-terminal region of TFIIB proteins and related factors. Residues of related proteins that are identical to those of *Arabidopsis* TFIIB1,2 proteins are highlighted in red, for cysteins of the zinc ribbon domain or in black. Residues of related proteins that are similar to those of *Arabidopsis* TFIIB1,2 are highlighted in gray. C) Summary of genes encoding B-type factors in eukaryotes. Boxes represent genes that can be identified in the genome of a given species.

**Figure 2:**
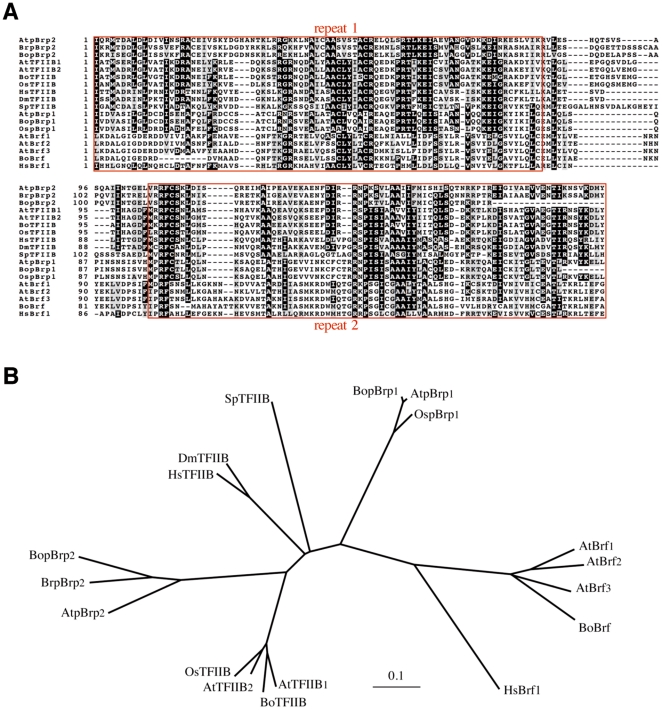
pBRP2 factors belong to the TFIIB family. A) Alignment of the core domain of pBRP2 proteins and B-type related factors. Repeats of the core domain are indicated by red boxes. Residues of related proteins that are identical to those of the Arabidopsis pBRP2 protein are highlighted in black, while similar residues are highlighted in gray. B) The phylogenetic tree was inferred from the core domain alignment. At, *Arabidopsis thaliana*; Bo, *Brassica oleracea*; Br, *Brassica rapa*; Os, *Oryza sativa*; Hs, *Homo sapiens*; Dm, *Drosophila melanogaster*; Sp, *Schizosaccharomyces pombe*.

A BLAST search [14] of the Arabidopsis genomic database using either TFIIB1 (At2g41630) or TFIIB2 (At3g10330) as query sequences revealed a putative TFIIB homolog (At3g29380), distinct from the pBRP1 variant (At4g36650) [12] and the three putative Arabidopsis BRF-type proteins (BRF1-3: At2g01280, At2g45100, and At3g09360) (Figure 1A). To determine whether At3g29380 gene was indeed expressed, we performed RT-PCR analysis on total RNAs from both leaves and flowers and found a product with the expected size only in flowers. Sequencing confirmed the annotation of the gene, harboring one intron and encoding a 337-amino acid (aa) TFIIB-related protein, hereafter named pBRP2 for plant-specific TFIIB-related protein 2 (Figures 1A and S1). A comparison of pBRP2 and eukaryotic TFIIB/BRF sequences confirmed the presence of two distinct domains that are characteristic of B-type factors: a conserved N-terminal zinc ribbon-containing domain (residues 1–118) which is 50% identical to TFIIB1 (Figures 1A and 1B), and a conserved C-terminal domain with two 80-aa imperfect direct repeats (residues 119–337) showing 40% identity with to TFIIB1 (Figures 1A and 2A). Interestingly, the region in which the pBRP2 sequence is most similar to TFIIBs is precisely within the N-terminal zinc ribbon that has been shown to interact with the RNAPII dock and the adjacent B-reader/-linker domains that are involved in DNA opening and transcription start site selection, respectively (Figure 1B) [5],[15].

To check whether pBRP2-like proteins are present in other species, we performed a TBLASTN search against the nucleotide databases with Arabidopsis pBRP2 as the query sequence. We identified *PBRP2* orthologues in the available, though incomplete, *Brassica rapa* (gb/AC189595; Figure S2A) and *Brassica oleracea* (NCBI: gnl/ti/104064696 ode19b06.b1; Figure S2B) genome databases, but not in the completed genomes of *Oryza sativa*, *Medicago truncatula* and *Populus trichocarpa* (Figure 1C). Moreover, no *PBRP2*-type gene could be detected in fungal or animal genomes. As expected, the deduced protein sequences of *PBRP2s* showed strong identity throughout their TFIIB-related region (with values ranging from 65% identity to 78% similarity) (Figure S2C). Sequence comparison of the pBRP2 core domain with those of plant TFIIB and BRF families indicates that pBRP2s are more related to TFIIB than to BRF (Figure 2A). Interestingly, most of the TFIIB core domain residues involved in promoter DNA and TBP interactions are conserved in pBRP2s, suggesting that the pBRP2 core domain has the ability to fold into a structure similar to the cyclin fold found in the TFIIB core domain [16]. To assess more precisely the relationship between pBRP2s and other B-type factors, the core domain sequence comparison was used to construct an unrooted phylogenetic tree, on which several clades of B-type factors could be identified (Figure 2B). Interestingly, all the newly identified pBRP2s and eukaryotic TFIIBs were grouped together in a clade that was distinct from the pBRP1 and the BRF families (Figure 2B). Taken together, our results indicate that pBRP2s define a plant-specific TFIIB-related protein subfamily and suggest that their distribution is restricted to members of the Brassicacea family, a sign of recent evolution in the history of land plants.

### *PBRP2* expression is restricted to reproductive organs and seeds

**Figure 3:**
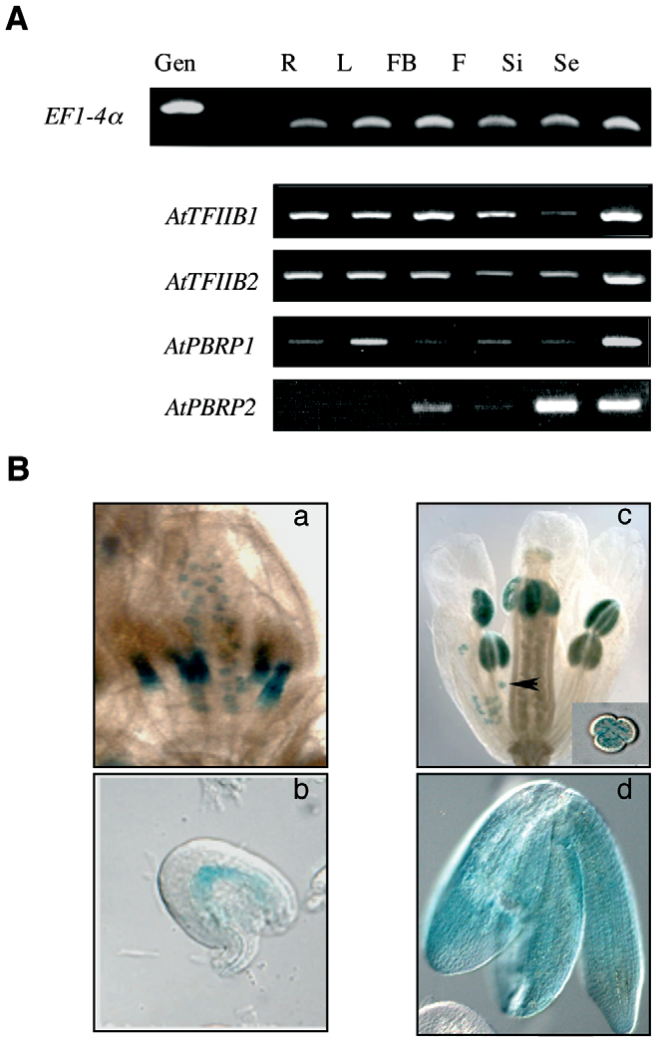
*AtPBRP2* is expressed in reproductive organs and seeds. A) RT-PCR analysis on total RNA prepared from roots (R), leaves (L), flower buds (FB), flowers (F), siliques (Si) and seeds (Se). Expression of *EF1-4*α is shown as loading control. B) Plants expressing the *PBRP2* promoter-GUS fusion show a GUS staining in anther filaments (Ba) and unfertilized ovules at stage 10–11 of flower development (Bb). Later, GUS staining is detected in anthers and especially in pollen grains (Bc, see black arrowhead and inset for GUS positive pollen grain), and finally, after fertilization, in embryos (Bd).

The expression pattern of Arabidopsis *PBRP2* was first analyzed by RT-PCR and a specific PCR product was detected in reproductive organs, including flower buds and siliques, as well as in seeds but not in the vegetative root and leaf organs (Figure 3A). These results indicate that the expression pattern of *PBRP2* is more restricted than those of the *TFIIBs* and *PBRP1* genes that are ubiquitously expressed in all organs tested (Figure 3A) [12]. To extend this analysis, five transgenic lines expressing a *PBRP2* promoter:*uidA* (GUS) fusion were examined at various stages of development. GUS expression patterns were spatially and temporally identical for all lines, showing good agreement with the RT-PCR data. Indeed, GUS staining could only be detected in the inflorescences (Figures 3Ba and 3Bc) and not in seedlings or mature leaves/roots (data not shown). At approximately floral stage 10–11 [17], the anther filaments and female gametophyte (the embryo sac) of unfertilized ovules stained strongly, although no obvious GUS staining was observed in the anthers at this stage (Figures 3Ba and 3Bb). We could not define a cell-specific pattern of GUS staining among the seven cells of the embryo sac, but GUS was unambiguously detected in the large central cell (Figure 3Bb). Staining became much stronger in the anthers at later stages of flower development, with strong staining in mature male gametophytes (Figure 3Bc, black arrowhead indicates a GUS positive pollen grain). No expression could be seen in the rest of the flower, including sepals, petals and stigma/style at any stage. A post-fertilization analysis indicated that *PBRP2* was also expressed in seeds (Figure 3Bd) after the torpedo stage of embryo development. GUS staining, however, was not detected at early stages of seed formation, from zygote to heart stage of embryo development.

**Figure 4:**
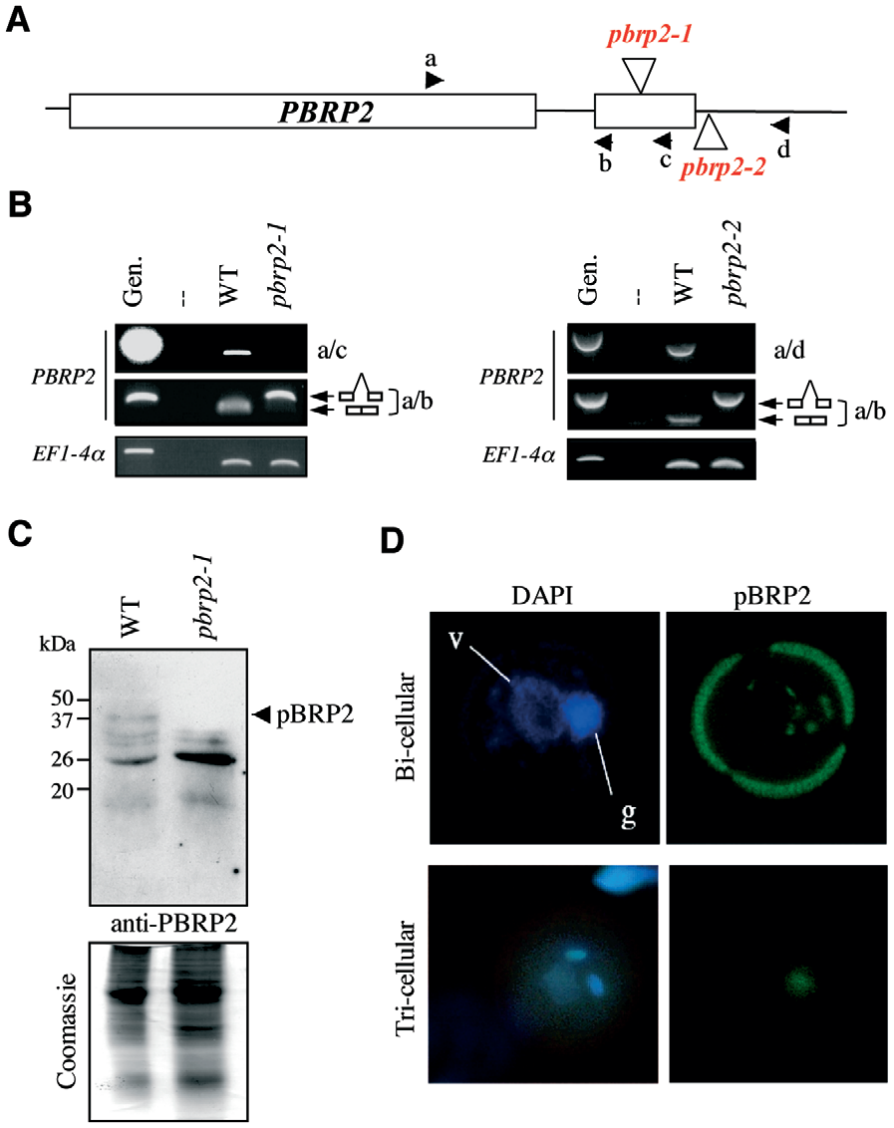
Identification of *pbrp2* knock-out lines. A) Schematic representation of the *AtPBRP2* gene. Exons are represented as boxes. Vertical arrowheads indicate T-DNA insertions and horizontal arrowheads the positions of primers used for RT-PCR analysis. B) RT-PCR performed to characterize *pbrp2*-1 and *pbrp2*-2 insertion mutants. Total mRNA was isolated from wild type and mutant flower buds. No full-length *PBRP2* transcripts are accumulated in mutants as revealed by amplification with primers a–c for *pbrp2*-1 and a–d for *pbrp2*-2. These mutant lines accumulate an unspliced truncated mRNA *PBRP2* transcript, as indicated by amplification using primers a–b and subsequent sequencing of the amplification product. Expression of *EF1-4*α is shown as loading control. – indicates a negative control with no DNA added. C) The pBRP2 protein is missing in *pbrp2*-1 pollen. Western analysis of protein extracts from pollen enriched fractions of wild type (WT) and mutant (*pbrp2-1*) plants probed with anti-pBRP2 antibody. The corresponding Coomassie blue staining is shown below. D) The pBRP2 protein localizes in pollen nuclei. Immunolocalization experiments suggest that pBRP2 is present in vegetative (v) and germinative (g) nuclei at the bicellular stage of pollen development and in vegetative nuclei at the tricellular stage. Stages of pollen maturation are indicated by DAPI staining. A strong signal is observed at the periphery of the bi-nucleate pollen grains, corresponding to autofluorescence from undefined components of the pollen wall. It is stronger in immature bi-nucleate pollen grains with 488 nm excitation, and becomes less intense in tri-nucleate pollen grains (see also Figure 3A).

To define more clearly the role of *PBRP2*, we raised peptide antibodies against a non-conserved region of pBRP2 (Figure S1 and Table S1) and characterized two Arabidopsis lines containing a T-DNA-disrupted mutant allele, *pbrp2*-1 and *pbrp2*-2 (Figure 4A). Plants homozygous for these mutant alleles were identified by PCR analysis, showing that *pbrp2* mutants are viable (data not shown). As expected for homozygous mutants, no full-length *PBRP2* transcript was detected by RT-PCR in *pbrp2*-1 and *pbrp2*-2 lines (Figure 4B; primers a–c and a–d, respectively). However, RT-PCR performed with upstream primers revealed the accumulation of an unspliced transcript in both *pbrp2* lines that probably results from a splicing defect due to the T-DNA insertion (Figure 4B; primers a–b). The retention of the intron introduces a premature stop codon, which at best could produce a potentially inactive pBRP2 truncated protein lacking *α*-helices BH4′-BH5′ of the second cyclin-type repeat of the core domain [16].

In order to assess the accumulation of pBRP2 protein *in vivo*, wild-type or *pbrp2-1* homozygous plants were used as controls in Western blot experiments. While no clear signal was obtained when flower buds were used as starting material (data not shown), the anti-pBRP2 antibody recognized a specific polypeptide of ∼38 kDa in wild-type but not *pbrp2*-1 purified pollen extracts (Figure 4C). Consistent with these results, anti-pBRP2 immunofluorescence labelling and DAPI staining of pollen grains at the bi-cellular stage revealed the presence of several bright spots located both in vegetative and generative nuclei (Figure 4D, top panel). Interestingly, at the tri-cellular stage, a more diffuse signal was seen in the vegetative nucleus, which was not detected in the generative nuclei (Figure 4D, bottom panel). This signal is pBRP2-specific since no nuclear staining by the fluorescent secondary antibody could be detected in *pbrp2*-1 pollen grains (Figure S3). Taken together, our data indicate that *PBRP2* encodes a plant-specific TFIIB-related factor whose expression is restricted to the male and female gametophytes, and late stages of seed development. To our knowledge, this is the first report of stage-specific expression for a TFIIB-type protein in eukaryotes. The dynamic pattern of *PBRP2* expression during flower development is somehow reminiscent of the germ cell/embryo-specific expressions of TBP-related factors (TRFs) in animals [9]–[11] and is suggestive of a specific role for pBRP2 during reproduction.

### pBRP2 does not contribute to RNAPIV/V activities in reproductive tissues

**Figure 5:**
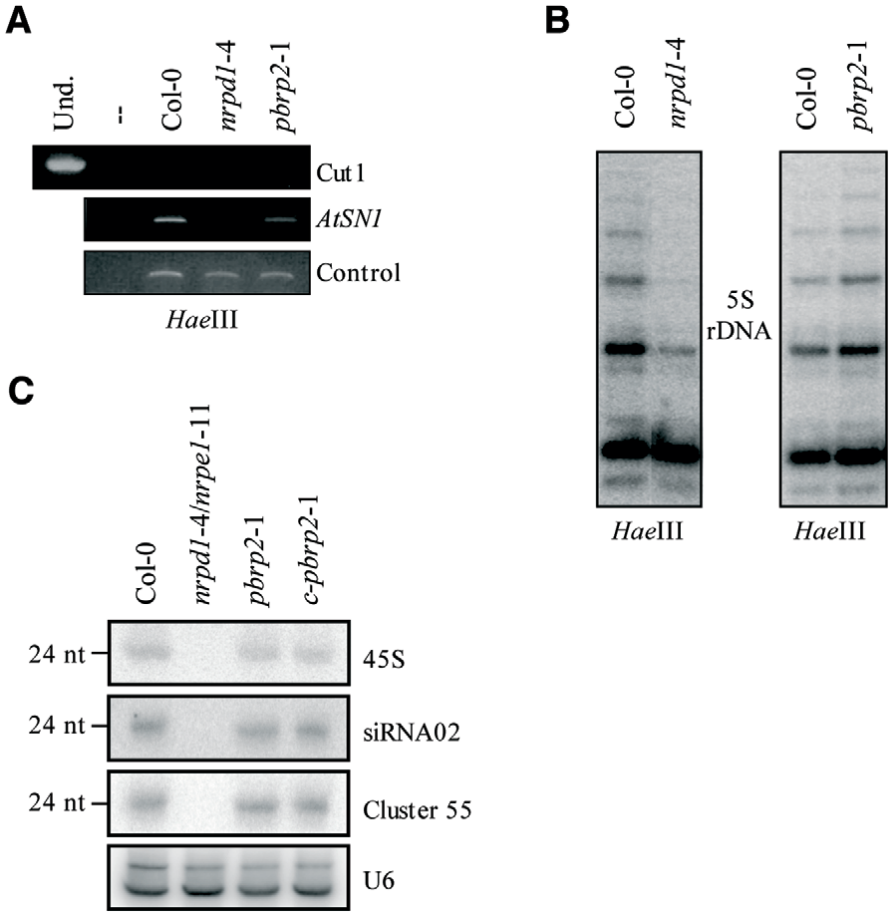
The PBRP2 factor is not required for RNAPIV/V activities. A) DNA methylation of *AtSN1* retroelement in *pbrp2*-1 and *nrpd1*-4 mutants. *Hae*III-digested genomic DNA was used as a template for PCR reactions using *AtSN1* and control primers. The cut1 primers would not amplify DNA if the digestion were complete. Und corresponds to undigested DNA. – indicates a negative control with no DNA added. B) Blot analysis of *5S* rDNA digested with methylation-sensitive restriction enzyme *Hae*III in *pbrp2*-1 and *nrpd1*-4 mutants and hybridized to a *5S* probe. C) Small RNA blot assays for U6, and three RNAPIV-dependent endogenous siRNAs in *nrpd1*-4/nrpe1-11 (mutant lacking both RNAPIV and RNAPV enzymes), *pbrp2*-1 and complemented *pbrp2*-1 mutant plants.

Several studies have recently implicated two forms of a plant-specific RNAPII-related enzyme, RNAPIV and RNAPV, in the activity of an siRNA-mediated chromatin silencing pathway, the RNA-directed DNA methylation (RdDM) pathway [18]–[21]. Although the activity of these enzymes in RdDM was initially though to be ubiquitous throughout plant development, recent data have demonstrated that most RNAPIV-dependent siRNAs are produced in the maternal gametophyte, culminating in the endosperm and early developping embryo [22]. These observations, together with our data concerning *PBRP2* gene evolution and its expression profile, prompted us to assess whether pBRP2 could be a plant-specific B-type factor dedicated to the functioning of RNAPIV and/or RNAPV in RdDM. To do so, we monitored the influence of *pbrp2*-1 together with *nrpd1*-4, a mutant lacking the largest subunit of RNAPIV (NRPD1), on the methylation status of *AtSN1* and *5S* rDNA, two known targets of the RdDM pathway. As expected from previous work [18]–[21], *AtSN1* and *5S* loci lost asymmetric methylation in *nrpd1*-4, where nearly complete cleavage with the methylation sensitive *Hae*III enzyme was observed (Figures 5A and 5B). In contrast to *nrpd1*-4, the *pbrp2*-1 mutant retained asymmetric methylation at both loci, as indicated by negligible digestion with *Hae*III (Figures 5A and 5B). In agreement with this observation, heterochromatic siRNA levels, which are a hallmark of RNAPIV action in developing endosperm, were not affected in the *pbrp2*-1 mutant, indicating that pBRP2 is not essential for RNAPIV-dependent siRNA production in the maternal gametophyte (Figure 5C). Taken together, these results indicate that pBRP2 does not contribute to RNAPIV/V activities in reproductive tissues and is unlikely to be an RNAPIV/V-associated B-type factor.

### *pbrp2* loss-of-function affects the development of the syncytial endosperm

**Figure 6:**
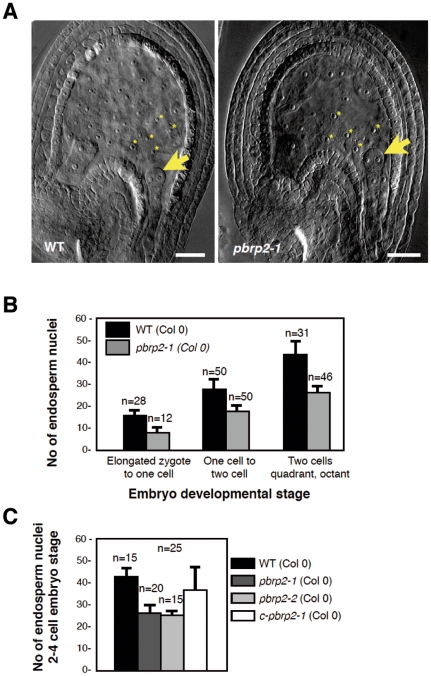
*pbrp2* endosperm phenotype and complementation. A) WT and mutant seeds at the 2–4 cell embryo stage (arrow). *pbrp2-1* mutant seeds are characterized by a reduced number of endosperm nuclei (stars) when compared to WT. Scale bar: 10 µm. B) Number of endosperm nuclei during seed development in *pbrp2-1* mutant and WT plants. C) Validation of the *pbrp2* phenotype. A second mutant allele, *pbrp2-2*, phenocopies the *pbrp2-1* allele. Complementation of the *pbrp2-1* mutation (*c-pbrp2-1*) restores the WT phenotype.

To determine the biological function of Arabidopsis pBRP2, we looked at the reproductive phenotype of the *pbrp2*-1 lines. Surprisingly, no obvious phenotype or lethality was detected during male and female gametophyte development, that is, in tissues where the gene is mostly expressed (data not shown). To further substantiate a potential defect in the *pbrp2*-1 male and female gametes, the transmission of T-DNA through each gamete were determined. To do so, the *PBRP2*/*pbrp2*-1 heterozygote mutant line was reciprocally backcrossed with wild-type and the number of plants for each of the two possible genotype outcomes was scored. If the mutant alleles were transmitted normally, the two genotypes would be represented equally. When the wild-type allele was used as the pollen donor, transmission of the mutant allele *pbrp2*-1 was normal, indicating that the loss of pBRP2 does not affect the female gametes (Table 1). Moreover, when the reciprocal cross was performed, transmission of the mutant allele *pbrp2*-1 was favored, suggesting that the loss of pBRP2 was not detrimental to pollen quality (Table 1). However, in the course of these experiments, we observed that *pbrp2-1* seeds were characterized by a significantly slower rate of proliferation during the syncytial phase of endosperm development when compared to wild-type. During that period the endosperm nuclei of wild-type plants undergo several rounds of nuclear divisions without cytokinesis, to form a syncytial structure containing up to 250 nuclei before cellularization (Figure 6A and Table 2) [23]. At different stages of embryo development, which appeared unaffected, we observed a ∼30% reduction of the number of endosperm nuclei (Figure 6B and Table 2). A similar phenotype was observed for the second mutant line, *pbrp2-2* (Figure 6C). Complementation of the *pbrp2-1* mutation using a full-length PBRP2 cDNA restored the wild-type phenotype (Figure 6C). Collectively, this indicates that the phenotype was a consequence of pBRP2 loss-of-function. Interestingly, the phenotype of pBRP2 inactivation is delayed compared with gene expression: it occurs only in the endosperm, while expression of the gene is prominent in the gametophytes.

**Table 1:** Genetic analysis of T-DNA transmissions.

Transmission	Cross	Gamete Frequency		Total	TE (a)
		pbrp2/pBRP2	pBRP2/pBRP2		
Female	WT×pbrp2/pBRP2	67	66	133	101 (<0.004)
Male	pbrp2/pBRP2×WT	112	94	206	119 (<0.78)

The heterozygous mutant line was reciprocally backcrossed with wild type and the number of plants per each of the two possible genotypic outcomes was scored on the basis of the antibiotic (Sulfadiazin (Sigma)) resistance conferred by the GABI T-DNA. The number of inferred gametes for each genotype is shown. Total: number of F1 plants scored.

**Table 2:** Endosperm nuclei counts in mutant and WT seeds.

Genotype	One cell, two cell embryo stage				Two cell, quadrant embryo stage			
	Mean +/− SD (n)	P values (Student)			Mean +/− SD (n)	P values (Student)		
		WT	pbrp2-1	pbrp2-2		WT	pbrp2-1	pbrp2-2
WT	26.6+/−3.2 (15)				40.8+/−3.6 (15)			
pbrp2-1	18.0+/−6.0 (30)	7.8 E-8			26.3+/−3.5 (20)	3.2 E-13		
pbrp2-2	18.1+/−2.3 (15)	4.5 E-9	0.5		25.6+/−1.8 (15)	1.1 E-12	0.2	
c-pbrp2-1	25.6+/−4,6(25)	0.2	1.1 E-6	2.5 E-8	38.1+/−6.8 (25)	0.05	2.7 E-9	6.6 E-10

SD: Standard Deviation; n: number of seeds analyzed.

To test for putative parent-of-origin effects, we reciprocally crossed *pbrp2-1* mutants with wild-type plants and examined endosperm development in F1 seeds. Interestingly, transmission of the mutant allele had a comparable effect on endosperm growth, irrespective of parental origin: the endosperm syncytium showed a significantly reduced number of nuclei whether the defective pBRP2 allele was paternally or maternally inherited (Table 3). This indicates that the presence of a wild-type allele in the endosperm is not sufficient to complement for the defective copy, consistent with the observation that the gene is not expressed post-fertilization. We thus propose that pBRP2 exerts both male and female gametophytic effects on endosperm proliferation.

**Table 3:** Parental effect analysis in the pbrp2-1 line.

Genotype	Mean +/− SD (n)	P values (Student)		
		WT×WT	pbrp2-1×pbrp2-1	WT×pbrp2-1
WT×WT	49.7+/−7.9 (7)			
pbrp2-1×pbrp2-1	37.6+/−12.0 (20)	3.9 E-3		
WT×pbrp2-1	40.9+/−10.4 (22)	1.6 E-2	0.2	
pbrp2-1×WT	39.0+/−5.9 (6)	8.7 E-3	0.3	0.3

All counts performed at the 2–4 cells embryo stage; SD: Standard Deviation; n: number of seeds analyzed.

To test for the impact of the mutation at later stages of seed development, we looked at the size of the cellularizing endosperm, when it reaches maximum size. In heterozygous plants, we observed a bimodal distribution of endosperm size, not found in wild type plants (Figures S4A and S4B), suggesting that loss of pbrp2 function resulted in significantly smaller final endosperm size. By contrast, measurement of mature seed size did not show any significant difference between mutant and wild type plants (Figure S4C), possibly as a consequences of compensatory mechanisms acting later during seed development.

### Conclusion

We have identified an additional TFIIB-related protein in plants, pBRP2, that is present in several Brassica species but not in more distant dicots or in monocots, suggesting that *PBRP2s* evolved around the origin of the crucifers that is estimated to be between 24–40 million years ago [24]. The restricted pattern of expression of pBRP2, which is similar to that of some TBP-related factors in animals, is unlike that of other B-factors characterized to date in eukaryotes, suggesting that it participates in a specific regulatory process. Given the high sequence similarity between TFIIBs and pBRP2s in the RNAPII-binding N-terminal domain [5], we propose that pBRP2 acts in an RNAPII-dependent regulatory pathway that is common to crucifers but absent or divergent in plants of evolutionarily distant origin.

The current data in Arabidopsis indicate that proliferation in the young endosperm is maternally and negatively controlled by the FERTILIZATION INDEPENDENT SEED Polycomb group complex, which regulates imprinting of target genes in the endosperm [25]. It is also dependent on yet undefined positive signals from the fertilized egg cell [26]. These results are often interpreted in the framework of the parental conflict theory, in which the parents have diverging interests in resource allocation to the seed: paternally expressed genes promote endosperm growth, and maternally expressed genes tend to inhibit endosperm growth [27]. Here we show that pBRP2 activity in both male and female gametophytes is important for nucleus proliferation during the syncytial stage of endosperm development, thus exerting positive bi-parental control. As a consequence of pBRP2-dependent transcription, progeny originating bi-parentally probably receive maximum resource allocation to the seed. This is reminiscent of the role of genomic imprinting in the mammalian embryo [28], which acts to enforce relatively strict bi-parental reproduction, and represents the first illustration of a converging strategy in plants.

## Materials and Methods

### Plant Genetic Analysis and DNA Constructs

After a 2 day-stratification at 4°C, plants were grown at 23°C with 16 h light on soil or under continuous light on Murashige and Skoog (MS) medium plates. The *pbrp2*-1 (GABI_736B11) and *pbrp2*-2 (GABI_204C11) lines were PCR-genotyped using 8409 and c or d primers respectively (all primers used are described in Table S1) [29]. The segregation of the wild-type *PBRP2* allele was tested using a–c and a–d primer pairs, respectively. For complementation experiments, the *PBRP2* genomic region region was PCR-amplified using primers pro*PBRP2*F/*PBRP2*R and cloned into a *Hind*III-*BamH*I digested 2×Flag-containing pCambia 1300 vector [30] using a *Hind*III-*Sal*I adapter. The pCambia 1300 derivative was introduced into the GV3121 strain of *Agrobacterium tumefaciens* and the *pbrp2*-1 mutant plants transformed by the floral dipping method. Transformed plants were screened on plates with MS medium containing hygromycin at 30 µg/l. For genetic analysis of T-DNA transmissions, wild-type and *pbrp2-1* homozygous plants were grown to maturity on soil and reciprocal crosses performed using five to eight flowers of each line. Genotypes of progeny from reciprocal crosses were determined on plates containing 7.5 mg/l Sulfadiazin (Sigma) on the basis of the antibiotic resistance conferred by the GABI T-DNA.

### Sequence analysis

Sequence alignment and phylogenetic analysis were derived using CLUSTAL W with default parameters as described in [31]. Database mining on the Arabidopsis genome sequence database, GSS, EST and nonredundant databases was by TBLASTN [14] alignment with the amino acid sequence of the *Arabidopsis* pBRP2 protein.

### RNA isolation and RT-PCR analysis

Total RNA was isolated from roots, leaves, flower buds and flower tissues of *pbrp2-1*, *pbrp2-2*, and wild-type *Arabidopsis* (ecotype Columbia) using the TRIzol reagent (Invitrogen) and from silique and seed tissues using an InViSorb kit (Invitek) according to manufacturer's instructions. After DNAse treatment, cDNA was obtained with an Affinity Multitemperature cDNA synthesis kit (Agilent) using an oligodT primer with 500 ng of RNA according to manufacturer's instructions. Semi-quantitative RT-PCR amplifications were done with primers 786/787 for *EF1-4α* as calibration control and primers 372/373, 549/550, 64/212 and 316/296 for *TFIIB1*, *TFIIB2*, *PBRP1* and *PBRP2* respectively.

### GUS staining

To insert the *PBRP2* promoter in front of the *uidA* (β-glucuronidase (*GUS*) reporter gene), a 990-bp PCR-amplified *Sal*I-*BamH*I promoter fragment (primers proPBRP2F-proPBRP2R) was ligated into the *Sal*I-*BamH*I sites of the promoter-less pBI101 plasmid (Clontech). The binary vector was transferred into the GV3121 strain of *Agrobacterium tumefaciens* to transform Arabidopsis wild-type plants by floral dip. Transformed plants were screened on plates with MS medium containing hygromycin at 30 mg/l. GUS staining was performed on stable transformants as previously described [12].

### Plant protein extraction, antibodies, and Western blotting

Pollen spores from Arabidopsis flower buds were purified as described previously [32]. After total protein extraction [12], pollen proteins were separated on SDS/PAGE gels and blotted onto PVDF membranes (Immobilon-P, Millipore). Rabbit antisera were raised against peptides designed in pBRP2, affinity-purified (Eurogentec), and used at a dilution of 1/1000.

### Genomic DNA extraction and methylation detection assays

Genomic DNA was extracted from seedlings using the Wizard Genomic DNA extraction kit (Promega). DNA was digested with *Hae*III restriction enzyme for *AtSN1* and *5S* analysis. PCR amplification was subsequently done on 150 ng of digested DNA using three pairs of primers. The control primers span a region lacking *Hae*III sites and are used to control equal template concentration. The cut1 primers flank a DNA sequence containing unmethylated *Hae*III sites and are used to check the completion of *Hae*III digestion. The *AtSN1* primers are used to monitor *AtSN1* methylation status. The *5S* Southern experiments were performed on 1 µg of genomic DNA digested by the methylation-sensitive enzyme *Hae*III and separated on 1% agarose gel. After blotting, hybridization was performed with a *5S* DNA probe.

### Small RNA extraction and analysis

Total RNA samples were extracted from inflorescences using TRIzol (Invitrogen). Thirty micrograms of total RNA were separated on a 15% polyacrylamide gel containing 7 M urea, electroblotted onto Hybond NX membranes (Amersham Pharmacia biotech Inc), and cross-linked with EDC (Sigma). Hybridization was performed in ULTRAhyb-Oligo Hybridization Buffer (Ambion) following the supplier's instructions. siRNA were detected using end-labeled DNA oligonucleotides.

### Immunolocalization

Stamens were fixed for 3 hours in 4% paraformaldehyde/1×PBS/2% Triton fixative, washed twice in 1×PBS, and dissected to isolate the pollen. Pollen was embedded in acrylamide and processed as described [33] with some modifications as followed. Samples were digested in an enzymatic solution (1% driselase; 0,5% cellulase; 1% pectolyase; 1% BSA; Sigma) for 25 min to 1 hour at 37°C, depending on the developmental stage, rinsed 3 times in 1×PBS and permeabilized for 1 to 2 hours in 1×PBS/2% Triton. They were then incubated overnight at 4°C with anti-pBRP2 antibody used at 1/100 dilution. The slides were washed day-long in 1×PBS/0,2% Triton, and coated overnight at 4°C with secondary antibodies (Alexa Fluor 488 conjugate, Molecular Probes) at 1∶400 dilution. After washing in 1×PBS/0,2% Triton for a minimum of 6 hours, the slides were incubated with DAPI (1 ug/ml in 1×PBS) for 1 hour, washed for 1 hour in 1×PBS, and mounted in PROLONG medium (Molecular Probes). Complete 3D pollen images were captured on a laser scanning confocal microscope (Leica SP2) equipped for DAPI (405 nm) and FITC (488 nm) excitations. Projections of selected optical sections were generated for this report, and edited using Graphic Converter (LemkeSOFT).

### Whole-mount ovule clearing

Siliques from *pbrp2-1*, *pbrp2-2* and wild-type plants were fixed in formalin/acetic acid/alcohol (FAA) reagent for 1 h. After dissection, seeds were cleared and mounted in Herr's medium. Preparations were observed using Nomarski optics with a Zeiss Axioplan microscope (Carl Zeiss AG, Germany).

## Supporting Information

Figure S1 **Primary sequence of the Arabidopsis pBRP2 protein.** The conserved cysteins of the amino-terminal zinc ribbon domain are underlined, as the imperfect direct repeats of the core domain. The peptide used for antibodies production is indicated in red.
(EPS)

Figure S2 **Structural features of** ***Brassica*** **orthologs of Arabidopsis pBRP2.** A) Primary sequence of the *Brassica rapa* pBRP2 protein. The conserved cysteins of the amino-terminal zinc ribbon domain are underlined, as are the imperfect direct repeats of the core domain. B) Primary sequence of the partial *Brassica oleracea* pBRP2 protein. The same code is used. C) Schematic representation and domain comparison of the pBRP2 proteins. The identity between the N-terminal and core domains of *Arabidopsis thaliana* pBRP2 and orthologues is indicated as a percentage.
(EPS)

Figure S3 **Antibody specificity controls.** A) Immunostaining of an anti-pBRP2 antibody in a *pbrp2*-1 homozygous line indicates high specificity of the antibody. A control antibody against Histone H3 acetylated lysine 9 (H3K9ac, in green) was used as a technical control. No signal above background level was detected for the anti-pBRP2 antibody (in red). B) Control images (without secondary antibody) of a bi-nucleate pollen grain shows that signal is observed at the periphery of the bi-nucleate pollen grains, corresponding to autofluorescence from undefined components of the pollen wall.
(EPS)

Figure S4 **Effect of pbrp2 loss-of-function on final endosperm and seed size.** A) Cellularizing endosperm in wild type plants. The surface used to calculate relative endosperm size is indicated by the dash line. The embryo is indicated by the arrowhead. B) Distribution of size measurements of cellularizing endosperms in wild-type (blue bars, n = 56) and heterozygous plants (green bars, n = 78). C) Distribution of size measurements of mature seeds in wild-type (blue bars, n = 106) and heterozygous plants (green bars, n = 95).
(EPS)

Table S1 **Primers and peptides used in this work.**
(DOC)

## References

[1] Orphanides G, Lagrange T, Reinberg D (1996). The general transcription factors of RNA polymerase II.. Genes Dev.

[2] Butler JE, Kadonaga JT (2002). The RNA polymerase II core promoter: a key component in the regulation of gene expression.. Genes Dev.

[3] Lagrange T, Kapanidis AN, Tang H, Reinberg D, Ebright RH (1998). New core promoter element in RNA polymerase II-dependent transcription: sequence-specific DNA binding by transcription factor IIB.. Genes Dev.

[4] Deng W, Roberts SGE (2007). TFIIB and the regulation of transcription by RNA polymerase II.. Chromosoma.

[5] Kostrewa D, Zeller ME, Armache KJ, Seizl M, Leike K (2009). RNA polymerase II-TFIIB structure and mechanism of transcription.. Nature.

[6] Reina JH, Hernandez N (2007). On a roll for new TRF targets.. Genes Dev.

[7] Schramm L, Pendergrast PS, Sun Y, Hernandez N (2000). Different human TFIIIB activities direct RNA polymerase III transcription from TATA-containing and TATA-less promoters.. Genes Dev.

[8] Muller F, Tora L (2004). The multicoloured world of promoter recognition complexes.. EMBO J.

[9] Zhang D, Penttila TL, Morris PL, Teichmann M, Roeder RG (2001). Spermiogenesis deficiency in mice lacking the Trf2 gene.. Science.

[10] Gazdag E, Santenard A, Ziegler-Birling C, Altobelli G, Poch O (2009). TBP2 is essential for germ cell development by regulating transcription and chromatin condensation in the oocyte.. Genes Dev.

[11] Müller F, Tora L (2010). TBP2 is a general transcription factor specialized for female germ cells.. J Biol.

[12] Lagrange T, Hakimi MA, Pontier D, Courtois F, Alcaraz JP (2003). Transcription factor IIB (TFIIB)-related protein (pBrp), a plant-specific member of the TFIIB-related protein family.. Mol Cell Biol.

[13] Imamura S, Hanaoka M, Tanaka K (2008). The plant-specific TFIIB-related protein, pBrp, is a general transcription factor for RNA polymerase I.. EMBO J.

[14] Altschul SF, Madden TL, Schäffer AA, Zhang J, Zhang Z (1997). Gapped BLAST and PSI-BLAST: a new generation of protein database search programs.. Nucleic Acids Res.

[15] Chen HT, Hahn S (2004). Mapping the location of TFIIB within the RNA polymerase II transcription preinitiation complex: a model for the structure of the PIC.. Cell.

[16] Nikolov DB, Chen H, Halay ED, Usheva AA, Hisatake K (1995). Crystal structure of a TFIIB-TBP-TATA-element ternary complex.. Nature.

[17] Smyth DR, Bowman JL, Meyerowitz EM (1990). Early flower development in Arabodopsis.. Plant Cell.

[18] Herr AJ, Jensen MB, Dalmay T, Baulcombe DC (2005). RNA polymerase IV directs silencing of endogenous DNA.. Science.

[19] Onodera Y, Haag JR, Ream T, Costa Nunes P, Pontes O (2005). Plant nuclear RNA polymerase IV mediates siRNA and DNA methylation-dependent heterochromatin formation.. Cell.

[20] Kanno T, Huettel B, Mette MF, Aufsatz W, Jaligot E (2005). Atypical RNA polymerase subunits required for RNA-directed DNA methylation.. Nature Genet.

[21] Pontier D, Yahubyan G, Vega D, Bulski A, Saez-Vasquez J (2005). Reinforcement of silencing at transposons and highly repeated sequences requires the concerted action of two distinct RNA polymerases IV in Arabidopsis.. Genes Dev.

[22] Mosher RA, Melnyk CW, Kelly KA, Dunn RM, Studhome DJ (2009). Uniparental expression of PolIV-dependent siRNAs in developing endosperm of Arabidopsis.. Nature.

[23] Boisnard-Lorig C, Colon-Carmona A, Bauch M, Hodge S, Doemer P (2001). Dynamic analyses of the expression of the HISTONE::YFP fusion protein in Arabidopsis show that syncytial endosperm is divided in mitotic domains.. Plant Cell.

[24] Blanc G, Hokamp K, Wolfe KH (2003). A recent polyploidy superimposed on older large-scale duplications in the Arabidopsis genome.. Genome Res.

[25] Baroux C, Pien S, Grossniklaus U (2007). Chromatin modification and remodeling during early seed development.. Curr Opin Genet Dev.

[26] Nowack MK, Shirzadi R, Dissmeyer N, Dolf A, Endl E (2007). Bypassing genomic imprinting allows seed development.. Nature.

[27] Haig D, Westoby M (2006). An earlier formulation of the conflict hypothesis of genomic imprinting.. Nature Genet.

[28] Surani MA, Barton SC, Norris ML (1987). Influence of parental chromosomes on spatial specificity in androgenetic-parthenogenetic chimaeras in the mouse.. Nature.

[29] Rosso MG, Li Y, Strizhov N, Reiss B, Dekker K (2003). An Arabidopsis thaliana T-DNA mutagenized population (GABI-Kat) for flanking sequence tag-based reverse genetics.. Plant Mol Biol.

[30] El-Shami M, Pontier D, Lahmy S, Braun L, Picart C (2007). Reiterated WG/GW motifs form functionally and evolutionarily conserved ARGONAUTE-binding platforms in RNAi-related components.. Genes Dev.

[31] Larkin MA, Blackshields G, Brown NP, Chenna R, McGettigan PA (2007). Clustal W and Clustal X version 2.0.. Bioinformatics.

[32] Honys D, Twell D (2004). Transcriptome analysis of haploid male gametophyte development in Arabidopsis.. Genome Biol.

[33] Bass HW, Marshall WF, Sedat JW, Agard DA, Cande WZ (1997). Telomeres cluster de novo before the initiation of synapsis: a three-dimensional spatial analysis of telomere positions before and during meiotic prophase.. J Cell Biol.

