# Supplementary material for: A Plant-Specific Transcription Factor IIB-Related Protein, pBRP2, Is Involved in Endosperm Growth Control
Source: PLoS One. 2011 Feb 24;6(2):e17216. doi: 10.1371/journal.pone.0017216 (PMC3044737; doi:10.1371/journal.pone.0017216)
Supplement: Table S1 — Primers and peptides used in this work. (DOC) [file pone.0017216.s005.doc]

Table S1

| **Primers used for PCR genotyping.** | |
| --- | --- |
| Name | Sequence |
| 8409 | 5’-atattgaccatcatactcattgc-3’ |
| a(322) | 5’-gcagagaatttcgatatccggag-3’ |
| c(340) | 5’-gtcgacactagttactgaaaattttgcagaatcccagg-3’ |
| d(855) | 5’-cgagactacaacaatcccaaagc-3’ |
| **Primers used for RT-PCR analysis.** | |
| Name | Sequence |
| a(322) | 5’-gcagagaatttcgatatccggag-3’ |
| b(507) | 5’-cacaacttccgcaacgatgcc-3’ |
| c(340) | 5’-gtcgacactagttactgaaaattttgcagaatcccagg-3’ |
| d(855) | 5’-cgagactacaacaatcccaaagc-3’ |
| 786 | 5’-ctaaggatggtcagacccg-3’ |
| 787 | 5’-cttcaggtatgaagacacc-3’ |
| 372 | 5’-ggggactggagcctggtcagtc-3’ |
| 373 | 5’-gcatatagaagaatcaagaaacgacttca-3’ |
| 549 | 5’-gcaagaagacaaaccacggacggtc-3’ |
| 550 | 5’-gagattagaagaggaagacactcaagg-3’ |
| 64 | 5’-aagtgtccgtactgttcat-3’ |
| 212 | 5’-caacagatggtcggaatgacca-3’ |
| 316 | 5’-ggcgattccagaagcggttgag-3’ |
| 296 | 5’-ttatactgaaaattttgcag-3’ |
| **Primers used to produce the promoter/PBRP2-GUS construct.** | |
| Name | Sequence |
| proPBRP2F | 5’-gtcgacctctatgataaaccatctcggc-3’ |
| PBRP2R | 5’-ggatcctactgaaaattttgcagaatcccagg-3’ |
| **Primers used for DNA methylation analysis.** | |
| Name | Sequence |
| ControlF | 5’-ggagagaggcttgttggatactgc-3’ |
| ControlR | 5’-gaacacgcatgacagtgggtggag-3’ |
| Cut1F | 5’-gaaggttctcatcatataccg-3’ |
| Cut1R | 5’-ggttttggaatgtttccaaccgc-3’ |
| AtSN1 | 5’-acttaattagcactcaaattaaacaaaataagt-3’ |
| AtSN1R | 5’-tttaaacataaraaraarttcctttttcatctac-3’ |
| **Primers used for siRNA analysis.** | |
| Name | Sequence |
| U6 | 5’-aggggccatgctaatcttctc-3 |
| 45S | 5’-gtctgttggtgccaagagggaaaagggctaat-3’ |
| siRNA02 | 5’-gttgaccagtccgccagccgat-3’ |
| Cluster55 | 5'-tttcccgcgtatatctctgcttgt-3’ |
| **Peptide used for antibody production.** | |
| Ab329 | CKYDGHANTKLRRGKK |
